# The association of estimated salt intake with blood pressure in a Viet Nam national survey

**DOI:** 10.1371/journal.pone.0191437

**Published:** 2018-01-18

**Authors:** Paul N. Jensen, Tran Quoc Bao, Tran Thi Thanh Huong, Susan R. Heckbert, Annette L. Fitzpatrick, James P. LoGerfo, Truong Le Van Ngoc, Ali H. Mokdad

**Affiliations:** 1 Department of Epidemiology, University of Washington, Seattle, WA, United States of America; 2 Department of Preventive Medicine, Viet Nam Ministry of Health, Hanoi, Viet Nam; 3 Department of Ethics and Social Medicine, Hanoi Medical University, Hanoi, Viet Nam; 4 Department of Global Health, University of Washington, Seattle, WA, United States of America; 5 Department of Medicine, University of Washington, Seattle, WA, United States of America; The University of Tokyo, JAPAN

## Abstract

**Objective:**

To evaluate the association of salt consumption with blood pressure in Viet Nam, a developing country with a high level of salt consumption.

**Design and setting:**

Analysis of a nationally representative sample of Vietnamese adults 25–65 years of age who were surveyed using the World Health Organization STEPwise approach to Surveillance protocol. Participants who reported acute illness, pregnancy, or current use of antihypertensive medications were excluded. Daily salt consumption was estimated from fasting mid-morning spot urine samples. Associations of salt consumption with systolic blood pressure and prevalent hypertension were assessed using adjusted linear and generalized linear models. Interaction terms were tested to assess differences by age, smoking, alcohol consumption, and rural/urban status.

**Results:**

The analysis included 2,333 participants (mean age: 37 years, 46% male, 33% urban). The average estimated salt consumption was 10g/day. No associations of salt consumption with blood pressure or prevalent hypertension were observed at a national scale in men or women. The associations did not differ in subgroups defined by age, smoking, or alcohol consumption; however, associations differed between urban and rural participants (p-value for interaction of urban/rural status with salt consumption, *p* = 0.02), suggesting that higher salt consumption may be associated with higher systolic blood pressure in urban residents but lower systolic blood pressure in rural residents.

**Conclusions:**

Although there was no evidence of an association at a national level, associations of salt consumption with blood pressure differed between urban and rural residents in Viet Nam. The reasons for this differential association are not clear, and given the large rate of rural to urban migration experienced in Viet Nam, this topic warrants further investigation.

## Introduction

While numerous epidemiological studies have reported an association between dietary salt intake and blood pressure, the majority of this evidence has come from developed countries.[1–3] Few studies on this topic have been conducted in developing countries, and those studies were focused on unique, geographically isolated populations with low levels of salt consumption.[2, 4–6] The impact of salt on blood pressure in developing countries with a high level of salt intake, such as those of South-east Asia, is unclear.[7, 8]

Viet Nam has undergone a period of rapid economic growth in the past 10–20 years, during which the country also experienced substantial rural to urban migration, increased tobacco use, the adoption of unhealthier diets, and decreased levels of physical activity.[8–12] These changes align with the “epidemiological transition,” the concept that as countries become more developed, the burden of disease shifts to chronic non-communicable diseases as the number of deaths from communicable diseases decreases and the average life expectancy increases.[13] Evidence from urban areas strongly suggests that the Vietnamese urban population is growing older and more obese, and that the prevalence of hypertension and diabetes is on the rise.[11, 12, 14, 15]

As a modifiable risk factor, salt consumption may be an appropriate target for public health interventions to lower population-wide blood pressure, which is hypothesized to lead to major improvements in public health.[1, 3, 16, 17] Although the cost of antihypertensive medications for an individual can be as little as pennies a day, salt reduction interventions are often cited as the most cost-effective means by which to lower population-wide blood pressure.[16, 18–20] Before any nation-wide salt reduction efforts are considered in Viet Nam, it is important to understand whether the effect of salt on blood pressure among Vietnamese is similar to that previously observed in developed countries.

Salt consumption is notoriously difficult to measure accurately, which has inhibited its investigation in resource-limited settings and developing countries.[3, 21] However, recent research has shown that a single spot urine collection can be used to provide useful estimates of salt intake in settings where multiple spot or 24-hour urine collections are not feasible.[22–27] We used spot urine sample data from a nationally representative population in Viet Nam to evaluate the association of salt intake with blood pressure and prevalent hypertension. We also assessed whether this association differed by age, smoking, alcohol consumption, or rural/urban residence.

## Methods

### Study population

The 2009 Viet Nam STEPwise approach to Surveillance (STEPS) survey is a cross-sectional study designed in accordance with World Health Organization (WHO) protocols to estimate the prevalence of key risk factors for non-communicable diseases among Vietnamese adults.[28] The 2009 Viet Nam STEPS design and recruitment are described in detail elsewhere.[29] Briefly, probability proportional to size sampling was used to select a nationally representative sample of 22,940 individuals aged 25–64 years from eight provinces, with each province representing a unique ecological region within Viet Nam. Between June and October 2009, trained interviewers conducted in-person interviews, and participants were invited to a clinic for a physical exam and blood and urine collection. A total of 14,706 Vietnamese adults completed an interview, physical examination, and blood collection; a spot urine sample was collected from a random subsample of 2,551 participants. Participants were excluded from this analysis if they reported acute illnesses or pregnancy, or if they reported current use of antihypertensive medications. This study was approved by the Viet Nam Ministry of Health Institutional Review Board, and participants provided written informed consent before participating.

### Data collection

Each province recruited a data collection team of approximately 20 local medical personnel who were trained by staff from the WHO, the Viet Nam Non-Communicable Disease office, and consultants from the Menzies Research Institute. Survey clinics were set up at each commune in a location convenient to participants, such as the People’s Committee Office (the local government administration office) or health center. The times that clinics opened were adjusted for each area based on the activities of local participants, and varied between 6 and 7AM. Participants attended the clinic after overnight fasting.

Urine and blood samples were collected before participants ate breakfast. Samples were collected in standard containers, and were refrigerated as they were transported to the Viet Nam National Institute of Nutrition, where they were kept at -20 degrees Celsius until analyzed. The concentrations of sodium and creatinine in the urine were measured using an ion selective electrode method. Fasting blood glucose and total cholesterol were measured from capillary whole blood using Roche Diagnostics Accutrend Plus glucometers.

At the clinic visit, participants were administered an in-person questionnaire by a study interviewer. The questionnaire was adapted from the WHO STEPS instrument (version 2.1) that was translated into Vietnamese.[28] Topics covered included demographic information, tobacco and alcohol use, physical activity, and medical history (self-reported history of hypertension, diabetes, and medication use).

Height, weight, and waist and hip circumference measurements were taken with the participant in bare feet without headwear or heavy clothing. Blood pressure was measured using an Omron HEM 907 digital automatic blood pressure monitor after the participants had rested for at least 15 minutes. Two blood pressure measurements were taken; if they differed by more than 25/15mmHg then a third measurement was taken. The average of the last two blood pressure measurements was used in the analysis. Once per week measurement tools and equipment were inspected by study staff and recalibrated if needed.

### Daily salt consumption estimation

Daily salt consumption was estimated from a fasting, mid-morning spot urine sample using a formula derived by Tanaka:[22]

$$\mathrm{eNa}=21.98\times {\left\{({\mathrm{Na}}_{S}/{\mathrm{Cr}}_{S})\times {\mathrm{Pr}.\mathrm{Cr}}_{24}\right\}}^{0.392}$$

eNa: Estimated 24-hour sodium excretion (mmol/day)
Na_S_: Sodium concentration in spot urine (mEq/L)
Cr_S_: Creatinine concentration in spot urine (mg/L)
Pr.Cr_24_: estimated 24hr urinary Cr excretion (mg/day)

$$\mathrm{Pr}.Cr_{24}=‑2.04\times Age+14.89\times Body\;weight\;\left(kg\right)+16.14\times Height\;\left(cm\right)‑2244.45$$

A validation study was conducted on a subsample of 154 participants between November and December 2010. Using the same collection protocol as described above, participants attended a study clinic after overnight fasting to provide mid-morning spot urine samples. At this point the participants began their 24-hour urine collections, and returned to the study clinic at the same time the following day to complete the 24-hour urine collection. After excluding eight incomplete or biologically implausible 24-hour urine samples (24-hour creatinine to body weight ratios that exceeded two standard deviations of the mean), spot urine based estimates of daily salt consumption were conservative (-12%; **S1 Fig**), but moderately correlated with 24-hour measured salt consumption (rho = 0.35).[30–32] In a sensitivity analysis, use of the Kawasaki formula had similar validity (rho = 0.34), but yielded higher estimated daily salt consumption than the Tanaka formula in this population (+15%; **S2 Fig**).[23]

Results are presented in terms of grams of salt intake (1 gram salt (sodium chloride) = 17.1mmol sodium). We excluded participants with estimated salt consumption levels that exceeded 3 standard deviations from the mean (less than 3 or more than 17 grams of salt per day).

### Statistical methods

Associations of daily salt consumption with systolic blood pressure were assessed using adjusted linear regression models. Relative risk regression was used to directly estimate the relative risk of hypertension associated with daily salt consumption, using generalized linear models with a Poisson distribution and robust standard errors.[33] Models included adjustment terms for age, sex, height, weight, smoking, total cholesterol, diabetes, and physical inactivity. Prevalent hypertension was defined as systolic blood pressure ≥140mmHg or diastolic blood pressure ≥90mmHg, and diabetes was defined as fasting glucose ≥126mg/dL or use of diabetes medication in the previous two weeks. Smoking was defined as current use of tobacco products; alcohol use was defined as five or more alcoholic drinks per week. Physical inactivity was defined as not meeting any of the following three criteria: 30 minutes of moderate-intensity physical activity on at least 5 days every week, 20 minutes of vigorous-intensity physical activity on at least 3 days every week, or a combination of vigorous- and moderate-intensity physical activity that exceeds 600 metabolic equivalent (MET)-minutes per week.[34] Rural and urban classification was based on the commune’s rural/urban designation in the 2009 national census.

To evaluate differences in the association of salt intake with blood pressure and prevalent hypertension by age (<45 years vs. ≥45 years), smoking (non-current vs. current), alcohol use (<5 drinks/week vs. ≥5 drinks/week), and rural/urban status, we tested interaction terms between salt intake and each of these risk factors in separate models. Interaction models included the primary set of adjustment covariates, except the age interaction model, which did not include a continuous term for age. Interaction models for smoking and alcohol consumption models were restricted to men due to the infrequency of reported smoking (2%) and alcohol use (<1%) among women. As a sensitivity analysis, we repeated analyses of salt intake with blood pressure and hypertension using Kawasaki and INTERSALT estimated salt consumption. [23, 35]

Analyses were conducted with STATA version 11.2 using the *svy* procedure. All analyses used STEPS sample weights that adjust for non-coverage and unequal probabilities of selection; sample weights incorporated post-stratification weights, which were calculated for each age (25–34, 35–44, 45–54, and 55–65 years) and sex stratum within each province using data from the 2009 Viet Nam national census.[36] Standard errors were computed using a robust variance estimator to take into account the complex sample design.

Of the 2,551 participants who provided spot urine samples, 7 were pregnant, 133 reported use of antihypertensive medications in the previous two weeks, 54 had missing body size or blood pressure measurements, and 24 had implausible salt intake levels and were excluded, leaving data from 2,333 participants available for analysis.

## Results

**Fig 1:**
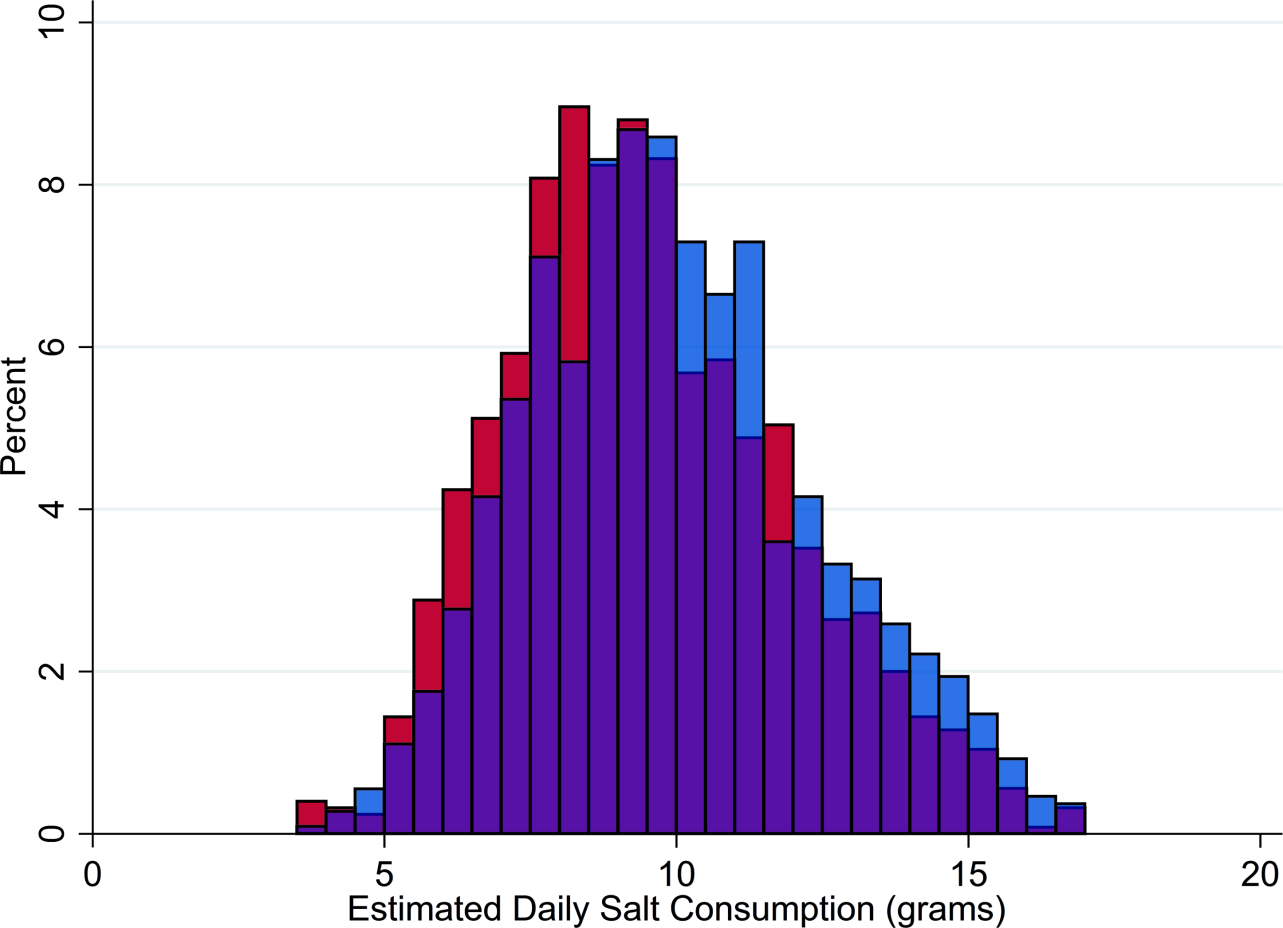
Daily salt intake. Blue = men. Red = women.

Characteristics of the 1,083 male and 1,250 female participants in the study are reported in **Table 1**. The average age of participants was 37 years and 11% had prevalent hypertension. On average, women were shorter, lighter, had lower blood pressure, and were less likely to smoke or drink alcohol than men. Participants consumed an average of 9.9 grams of salt per day, the distribution of which was relatively normal (**Fig 1**); 99% consumed more than the WHO recommended limit of 5 grams of salt per day.[37]

**Table 1:** Characteristics of 1,083 male and 1,250 female 25–64 year old participants in the 2009 Viet Nam STEPS survey.*

	Men		Women	
	Mean or %	SE	Mean or %	SE
Age (years)	37	0.34	37	0.45
Height (cm)	163	0.38	153	0.31
Weight (kg)	59	1.06	50	0.46
BMI (kg/m^2^)	22	0.36	21	0.15
Current smoker	59%	0.04	2%	0.01
≥ 5 Alcoholic drinks/week	15%	0.02	<1%	0.00
Physically inactive	32%	0.03	39%	0.04
Urban residence	31%	0.02	34%	0.02
Kinh ethnicity	97%	0.01	98%	0.00
Systolic BP (mmHg)	123	0.95	113	0.88
Diastolic BP (mmHg)	75	0.80	70	0.67
Hypertension	15%	0.02	5%	0.01
Fasting Glucose (mmol/L)	4.2	0.11	3.9	0.05
Diabetes	12%	0.03	9%	0.02
Total Cholesterol (mmol/L)	4.7	0.05	4.7	0.05
Estimated salt intake (g/day)	10.2	0.19	9.5	0.15

*Nationally representative; weighted with sampling and post-stratification weights

In sex-stratified models adjusted for age, height, weight, smoking, total cholesterol, diabetes, and physical inactivity, there was no evidence of an association of salt consumption with systolic blood pressure or prevalent hypertension (**Table 2**).

**Table 2:** Sex-stratified regression models of salt intake (g/day) with systolic blood pressure and prevalent hypertension.

	Minimally Adjusted Model*		Primary Model**	
		95% CI		95% CI
Systolic Blood Pressure (mmHg)				
Men	β = -0.20	-1.03, 0.63	β = -0.12	-0.83, 0.58
Women	β = 0.29	-0.26, 0.84	β = -0.06	-0.73, 0.61
Hypertension***				
Men	RR = 0.94	0.79, 1.12	RR = 0.97	0.87, 1.09
Women	RR = 0.98	0.88, 1.09	RR = 0.92	0.83, 1.02

*Includes age and body mass index as adjustment covariates

**Includes age, height, weight, smoking, total cholesterol, diabetes, and physical inactivity as adjustment covariates

***Systolic blood pressure ≥ 140mmHg or diastolic blood pressure ≥ 90mmHg

There was no evidence of an association of salt consumption with systolic blood pressure or prevalent hypertension in subgroups defined by age, smoking, alcohol consumption, or urban/rural residence (**Table 3**). Although we did not observe statistically significant associations of salt consumption with blood pressure within either urban or rural subgroups, there was evidence that these associations differed (p-value for interaction of urban/rural status with salt consumption, *p* = 0.02), which suggested that higher salt consumption may be associated with higher systolic blood pressure in urban participants but lower blood pressure in rural participants. While the mean systolic blood pressure was similar in urban and rural participants (119 vs. 118 mmHg, *p* = 0.79), salt consumption levels were slightly lower in urban residents than in rural residents (9.5 vs 10.1 g/day, *p* = 0.01). Restricting analyses to Kinh participants, using the Kawasaki or INTERSALT formulas to estimate salt intake, and including salt consumption levels that exceeded three standard deviations from the mean did not alter any of these findings (**S3 Fig; S1, S2 and S3 Tables)**.

**Table 3:** Age-, smoking-, alcohol-, and rural/urban-stratified adjusted* regression models of salt intake (g/day) with systolic blood pressure and prevalent hypertension.

		Systolic Blood Pressure				Hypertension			
	Mean Salt Intake (g/day)	Mean (mmHg)	β	95% CI	p**	Prevalence	RR	95% CI	p**
Age***									
<45 years	10.0	117	-0.08	-0.67, 0.50		9%	0.96	0.85, 1.10	
≥45 years	9.6	124	0.15	-0.52, 0.81	0.58	18%	0.97	0.89, 1.06	0.94
Current smoker****									
No	10.3	124	-0.04	-0.85, 0.77		15%	0.99	0.82, 1.20	
Yes	10.2	123	-0.30	-1.26, 0.66	0.76	16%	0.97	0.85, 1.11	0.95
Alcohol consumption****									
<5 drinks/week	10.3	122	0.18	-0.56, 0.93		14%	1.00	0.88, 1.14	
≥5 drinks/week	9.7	129	-0.49	-1.46, 0.48	0.46	23%	1.07	0.92, 1.24	0.71
Place of residence									
Rural	10.1	118	-0.41	-0.99, 0.17		10%	0.92	0.82, 1.02	
Urban	9.5	119	0.59	-0.29, 1.46	0.02	12%	1.01	0.86, 1.18	0.29

*Includes adjustment terms for age, sex, height, weight, smoking, total cholesterol, diabetes, and physical inactivity

**p-value for interaction

***Continuous age not included as an adjustment term

****Analyses restricted to men-only

## Discussion

We observed that salt consumption was not associated with systolic blood pressure or the risk of prevalent hypertension at a national level in Viet Nam. We also observed that associations with systolic blood pressure differed between urban and rural participants, suggesting that higher salt consumption may be associated higher systolic blood pressure in urban residents but lower systolic blood pressure in rural residents in Viet Nam. However, associations in each subgroup were not statistically significant and may be due to chance.

To the best of our knowledge, this is the first study to examine the association of salt consumption with blood pressure at a national level in South-east Asia.[3, 7, 8] The national average estimated salt consumption level we observed (9.9g/day) was in line with levels recently reported in rural areas near Hanoi (8.5g/day to 10.8g/day).[38, 39] Our finding of no association of salt consumption with blood pressure at a national level contrasts with a recent meta-analysis of thirty-four trials in developed countries, which observed a positive association of salt intake with blood pressure (difference in systolic blood pressure (mmHg) per one-gram higher level of salt consumption; β = 0.95, 95%CI: 0.72, 1.18).[1] A recent South Korean national survey estimated daily sodium excretion from spot urine samples and reported an association of salt consumption with higher blood pressure; however, this study did not collect information on antihypertensive medication use and was not able to account for this in its analysis.[40] We did not observe the differential association of salt consumption with systolic blood pressure or hypertension by smoking status, alcohol consumption, or age that has been observed elsewhere.[2]

Reasons that salt consumption may have a different impact on the blood pressure of urban participants than of rural participants are not clear. The varying degree to which an individual’s blood pressure responds to changes in salt intake (“salt-sensitivity”) is largely driven by renal function.[41] A number of genetic factors have been found to impair renal function and are associated with salt-sensitivity.[42–45] Both rural and urban residents were predominantly Kinh ethnicity (98% and 96%, respectively; *p* = 0.23), and restricting analyses to Kinh participants did not alter our findings. Given that the rate of rural to urban migration from 1999 to 2010 increased by an average of 9.2% per year, and one-sixth of the urban population in 2009 had moved from rural areas in the past five years,[46] it would seem unlikely that genetic differences between rural and urban participants fully explain the stronger association of salt consumption with blood pressure in urban residents.

Apart from genetics, a number of other factors are associated with salt-sensitivity, including old age and diets low in potassium or calcium. [41, 42, 47–49] While we were able to adjust for age as a confounding variable, we were not able to adjust for dietary potassium or calcium, or for markers of renal function, as they were not measured in this survey. If these factors were more common among urban participants than rural participants, they could impair renal function (thereby increasing the salt-sensitivity) of urban residents more than rural residents, which could help explain the differential association of salt consumption with blood pressure by urban/rural status that we observed.

It is also possible that because rural participants were more likely to work in agriculture, they would lose more salt in sweat than urban participants due to the physical nature of their work and the hot climate of Viet Nam. Although there is a common perception that extra salt should be consumed to replace electrolytes lost due to perspiration, the amount of salt needed to maintain electrolyte balance in most conditions is actually quite low (approximately 0.6–1.2 grams per day for an average adult), so the amount consumed in this population far exceeded physiological need.[3, 50, 51] While some sodium is indeed secreted through perspiration, the amount is relatively small (0.6–4.1 grams per liter of sweat), with heat acclimated people on the low end of that spectrum.[21] Because the study population would be well acclimated to the heat and humidity, and because we adjusted for physical activity in our analyses, we do not have reason to believe that differential perspiration loss by rural/urban status influenced our findings.

Although many studies have documented a rise in blood pressure corresponding with rural to urban migration, few have examined the influence of salt on this association.[52, 53] A 1991 study of the Yi, an ethnic minority in southwestern China, collected blood pressure measurements and 24-hour urine samples from Yi farmers who lived in the rural mountains and Yi who had migrated to nearby urban centers.[4] Rural Yi farmers had low levels of salt consumption (5.6g/day) and one of the lowest average blood pressures in the world (98/60mmHg); both of which were lower than those of Yi urban migrants (9.3g/day, 107/69mmHg).[2] The study authors concluded that, due to the ethnic similarity of the urban migrants to the rural farmers, the higher blood pressure observed in the urban migrants was largely due lifestyle changes, including increased salt intake. A 1984 study of Kenyan rural to urban migrants provided similar findings.[5] In our population, rural participants had a slightly higher average level of salt consumption than urban participants, but the mean systolic blood pressure was similar in the two groups.

The cross-sectional nature of the study is a limitation of this analysis. Because we cannot assess whether salt consumption levels predated systolic blood pressure levels, we are only able to assess the correlation of salt consumption with blood pressure and prevalent hypertension, rather than causality. Another limitation is our use of an imperfect measure of salt consumption. Although 24-hour urine collection is the gold standard for estimating salt consumption in epidemiological studies, spot urine sample-derived estimates are a useful measure of salt consumption and are gaining popularity in large-scale epidemiological studies.[24, 26] Further, the levels of estimated daily salt consumption we observed were similar to those reported in region-specific studies of salt consumption in Viet Nam.[38, 39] However, deriving daily salt consumption from a single spot urine often results in overestimates among those at lower salt consumption levels and underestimates among those at higher salt consumption levels.[25, 30, 54] In our population where 99% consumed more than the WHO recommended limit of salt per day, use of spot urine may have underestimated daily salt consumption and impaired our ability to detect associations with blood pressure and hypertension, resulting in conservative estimates of the association of salt consumption with blood pressure and prevalent hypertension or an apparent lack of association where one may truly be present.[55] Finally, the relatively young upper age (64 years) of included participants inhibited our ability to assess associations among those at greatest risk of high blood pressure.

## Conclusions

There was no evidence of an association of salt consumption with elevated systolic blood pressure at a national level in Viet Nam; however, the association of salt consumption with blood pressure differed in urban versus rural residents. The reasons for this differential association are not clear, and given the large rate of rural to urban migration experienced in Viet Nam, this topic warrants further investigation.

## Supporting information

S1 Fig Bland-Altman plot, Tanaka estimated vs. 24-hour urine measured salt consumption in spot urine validity subset population (N = 146). (TIF)

S2 Fig Bland-Altman plot, Kawasaki estimated vs. 24-hour urine measured salt consumption in spot urine validity subset population (N = 146). (TIF)

S3 Fig Average daily salt consumption estimated from a spot urine sample using three formulas. Blue = men. Red = women.
(TIF)

S1 Table Sex-stratified regression models of Kawasaki and INTERSALT estimated salt intake (g/day) with systolic blood pressure and prevalent hypertension. (DOCX)

S2 Table Age-, smoking-, alcohol-, and rural/urban-stratified adjusted* regression models of Kawasaki and INTERSALT estimated salt intake (g/day) with systolic blood pressure and prevalent hypertension. (DOCX)

S3 Table Sex-stratified regression models* of estimated salt intake (g/day) with systolic blood pressure and prevalent hypertension, with and without outlier** exclusion. (DOCX)

S1 Dataset Spot urine validity dataset. (CSV)

S2 Dataset Analytic dataset. (CSV)
