# Supplementary figures and images for: The association of estimated salt intake with blood pressure in a Viet Nam national survey
Source: PLoS One. 2018 Jan 18;13(1):e0191437. doi: 10.1371/journal.pone.0191437 (PMC5773206; doi:10.1371/journal.pone.0191437)

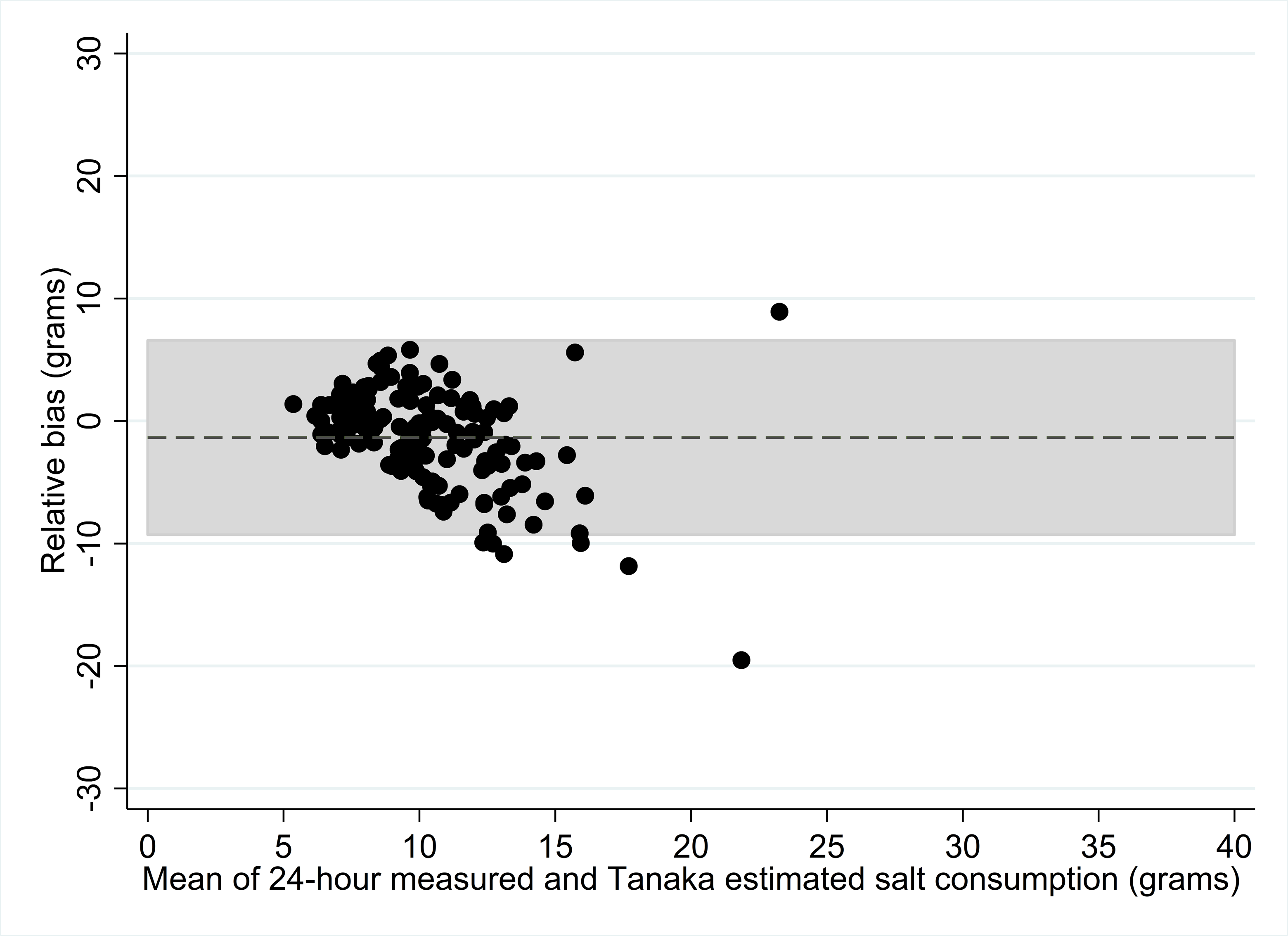

Supplement: S1 Fig — (TIF) [file pone.0191437.s001.tif]

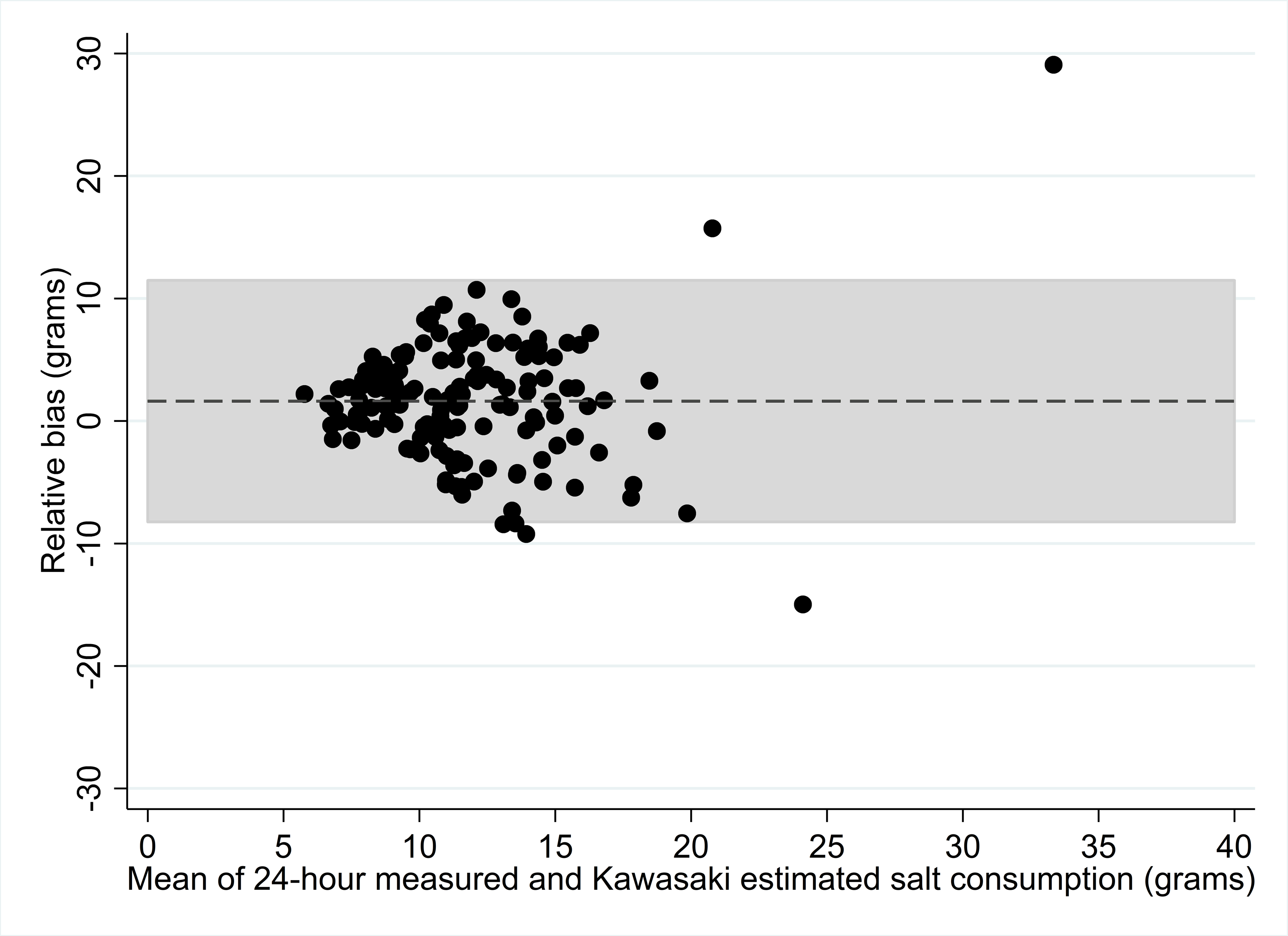

Supplement: S2 Fig — (TIF) [file pone.0191437.s002.tif]

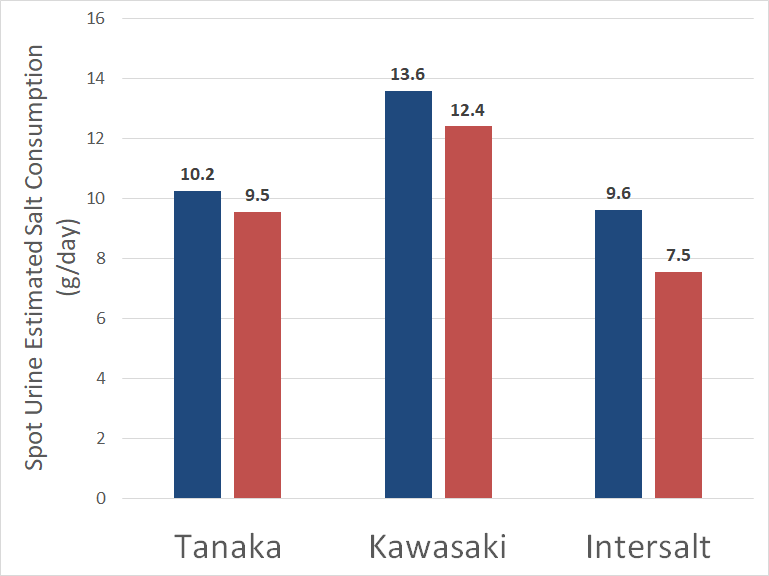

Supplement: S3 Fig — Blue = men. Red = women. (TIF) [file pone.0191437.s003.tif]
