# Supplementary material for: The association of estimated salt intake with blood pressure in a Viet Nam national survey
Source: PLoS One. 2018 Jan 18;13(1):e0191437. doi: 10.1371/journal.pone.0191437 (PMC5773206; doi:10.1371/journal.pone.0191437)
Supplement: S1 Table — (DOCX) [file pone.0191437.s004.docx]

**Supplemental Table 1.** **Sex-stratified regression models of Kawasaki and INTERSALT estimated salt intake (g/day) with untreated systolic blood pressure and prevalent hypertension**

|  | Kawasaki | | | | |  | INTERSALT | | | | |
| --- | --- | --- | --- | --- | --- | --- | --- | --- | --- | --- | --- |
|  | Minimally adjusted  model* | |  | Primary Model** | |  | Minimally adjusted  model* | |  | Primary Model** | |
|  |  | 95% CI |  |  | 95% CI |  |  | 95% CI |  |  | 95% CI |
| Systolic Blood Pressure  (mmHg) |  |  |  |  |  |  |  |  |  |  |  |
| Men | β = -0.12 | -0.61, 0.38 |  | β = -0.07 | -0.50, 0.35 |  | β = -0.06 | -1.21, 1.08 |  | β = 0.12 | -0.79, 1.03 |
| Women | β = 0.18 | -0.15, 0.51 |  | β = -0.05 | -0.45, 0.36 |  | β = 0.17 | -0.93, 1.27 |  | β = 0.29 | -0.88, 1.47 |
| Hypertension*** |  |  |  |  |  |  |  |  |  |  |  |
| Men | RR = 0.97 | 0.87, 1.07 |  | RR = 0.98 | 0.92, 1.05 |  | RR = 0.91 | 0.71, 1.17 |  | RR = 0.93 | 0.82, 1.06 |
| Women | RR = 0.98 | 0.92, 1.05 |  | RR = 0.94 | 0.89, 1.01 |  | RR = 1.02 | 0.84, 1.23 |  | RR = 1.02 | 0.84, 1.26 |

*Includes age and body mass index as adjustment covariates

**Includes age, height, weight, smoking, total cholesterol, diabetes, and physical inactivity as adjustment covariates

***Systolic blood pressure ≥ 140mmHg or diastolic blood pressure ≥ 90mmHg
