# Supplementary material for: The association of estimated salt intake with blood pressure in a Viet Nam national survey
Source: PLoS One. 2018 Jan 18;13(1):e0191437. doi: 10.1371/journal.pone.0191437 (PMC5773206; doi:10.1371/journal.pone.0191437)
Supplement: S2 Table — (DOCX) [file pone.0191437.s005.docx]

**Supplemental Table 2.** **Age-, Smoking-, Alcohol-, and Rural/Urban-stratified adjusted* regression models of Kawasaki and INTERSALT estimated salt intake (g/day) with untreated systolic blood pressure and prevalent hypertension**

|  | Kawasaki | | | | | | |  | INTERSALT | | | | | | |
| --- | --- | --- | --- | --- | --- | --- | --- | --- | --- | --- | --- | --- | --- | --- | --- |
|  | Linear - Systolic | | |  | Logistic - Hypertension | | |  | Linear - Systolic | | |  | Logistic - Hypertension | | |
|  | β | 95% CI | *p*** |  | RR | 95% CI | *p*** |  | β | 95% CI | *p*** |  | RR | 95% CI | *p*** |
| Age*** |  |  |  |  |  |  |  |  |  |  |  |  |  |  |  |
| <45 years | -0.05 | -0.40, 0.31 |  |  | 0.98 | 0.90, 1.05 |  |  | -0.15 | -1.06, 0.77 |  |  | 0.91 | 0.77, 1.08 |  |
| ≥45 years | 0.07 | -0.32, 0.47 | 0.62 |  | 0.98 | 0.93, 1.04 | 0.92 |  | 0.95 | -0.09, 1.98 | 0.17 |  | 1.00 | 0.86, 1.15 | 0.77 |
| Current smoker**** |  |  |  |  |  |  |  |  |  |  |  |  |  |  |  |
| No | -0.01 | -0.49, 0.48 |  |  | 1.00 | 0.89, 1.12 |  |  | 0.38 | -1.03, 1.8 |  |  | 0.99 | 0.75, 1.30 |  |
| Yes | -0.19 | -0.76, 0.38 | 0.71 |  | 0.98 | 0.91, 1.06 | 0.99 |  | -0.10 | -1.23, 1.03 | 0.46 |  | 0.92 | 0.79, 1.08 | 0.55 |
| Alcohol consumption**** |  |  |  |  |  |  |  |  |  |  |  |  |  |  |  |
| <5 drinks/wk | 0.11 | -0.34, 0.55 |  |  | 1.00 | 0.93, 1.08 |  |  | 0.37 | -0.68, 1.42 |  |  | 0.94 | 0.80, 1.10 |  |
| ≥5 drinks/wk | -0.29 | -0.85, 0.28 | 0.44 |  | 1.04 | 0.95, 1.13 | 0.69 |  | -0.60 | -1.94, 0.74 | 0.45 |  | 0.98 | 0.80, 1.20 | 0.35 |
| Place of residence |  |  |  |  |  |  |  |  |  |  |  |  |  |  |  |
| Rural | -0.25 | -0.60, 0.09 |  |  | 0.95 | 0.89, 1.01 |  |  | -0.15 | -0.96, 0.66 |  |  | 0.87 | 0.77, 0.99 |  |
| Urban | 0.36 | -0.17, 0.89 | 0.02 |  | 1.01 | 0.92, 1.11 | 0.26 |  | 0.95 | -0.49, 2.38 | 0.05 |  | 1.04 | 0.86, 1.27 | 0.22 |

*Includes adjustment terms for age, sex, height, weight, smoking, total cholesterol, diabetes, and physical inactivity

**p-value for interaction

***Continuous age not included as an adjustment term

****Analyses restricted to men-only
