# Supplementary material for: The association of estimated salt intake with blood pressure in a Viet Nam national survey
Source: PLoS One. 2018 Jan 18;13(1):e0191437. doi: 10.1371/journal.pone.0191437 (PMC5773206; doi:10.1371/journal.pone.0191437)
Supplement: S3 Table — (DOCX) [file pone.0191437.s006.docx]

**Supplemental Table 3. Sex-stratified regression models* of estimated salt intake (g/day) with systolic blood pressure and prevalent hypertension, with and without outlier** exclusion**

|  | Outliers** excluded | |  | All participants | |
| --- | --- | --- | --- | --- | --- |
|  |  | 95% CI |  |  | 95% CI |
| Systolic Blood Pressure (mmHg) |  |  |  |  |  |
| Men | β = -0.12 | -0.83, 0.58 |  | β = -0.10 | -0.79, 0.59 |
| Women | β = -0.06 | -0.73, 0.61 |  | β = -0.07 | -0.56, 0.43 |
| Hypertension*** |  |  |  |  |  |
| Men | RR = 0.97 | 0.87, 1.09 |  | RR = 0.97 | 0.87, 1.07 |
| Women | RR = 0.92 | 0.83, 1.02 |  | RR = 0.91 | 0.83, 1.01 |

*Models include adjustment for age, height, weight, smoking, total cholesterol, diabetes, and physical inactivity

**Salt consumption of less than 3 or greater than 17 grams per day

***Systolic blood pressure ≥ 140mmHg or diastolic blood pressure ≥ 90mmHg
